# Editorial: Autoimmune diseases in childhood

**DOI:** 10.3389/fimmu.2024.1516530

**Published:** 2024-11-14

**Authors:** Bernadete L. Liphaus, Jocelyne Demengeot, Magda Carneiro-Sampaio

**Affiliations:** ^1^ Laboratory of Medical Investigation, Faculdade de Medicina, Universidade de SãoPaulo, São Paulo, Brazil; ^2^ Instituto Gulbenkian de Ciência, Oeiras, Portugal

**Keywords:** autoimmune diseases, childhood, early-onset, inborn errors of immunity (IEI), next-generation sequencing (NGS)

The field of autoimmune diseases in childhood presents challenges to physicians regarding diagnosis, monitoring, and treatment (1–3). Didactically, autoimmune diseases can be classified as organ-specific or multi-system organ involvement (1–3). Despite increasing research, the exact cause of autoimmune disorders is not entirely understood (1–3). The main feature of autoimmune diseases is immune dysregulation which leads to immune-mediated damage of healthy tissues and cells and results from the breakdown of the central or peripheral tolerance (1–4). Autoimmune diseases in childhood are often less extensively studied (1–3). In this regard, understanding how autoimmune responses initiate and progress will allow the creation of novel therapeutics and improve the diagnoses and management strategies of these diseases (1–3).

Autoimmune diseases in childhood, in particular, those of early onset (diagnosed at < 5 years of age), have been linked to a series of genetic determinants, including human leukocyte antigen (HLA) and non-HLA variants as well as gene variants related to inborn errors of immunity (5–7).

Inborn errors of immunity (IEI) are genetic disorders that may manifest as increased susceptibility to infections, autoinflammatory, allergic, or malignant diseases, however, there are few IEI in which an autoimmune manifestation has never been observed (6). Regarding this, next-generation sequencing (NGS) has uncovered an array of genetic explanations for autoimmune diseases (6, 8). Genetic variants can alter various cellular functions, and the relative risk of each in the disease phenotype’s final effect can vary greatly (2, 6). An example is the LRBA deficiency which may manifest as a common variable immune deficiency (CVID) as well as inflammatory bowel disease, endocrinopathies, autoimmune hemolytic anemia, thrombocytopenia, arthritis, and systemic lupus erythematosus (8).

The challenge of unveiling the pathogenesis of autoimmune diseases in childhood is highlighted in the 23 interesting research articles collected from the 58 total manuscripts submitted under this Research Topic “*Autoimmune Diseases in Childhood*”, of the Frontiers in Immunology.

The power of genetic variants to drive autoimmune diseases, even in different ethnicities, is specially revealed in patients with early-onset disease, for example, in the study of Caramalho et al. which shows the discriminative factor of haplotype DRB1*04:08-DQ8 in the early development of type 1 diabetes. On the opposite side, Dashti et al.’s work demonstrates the power of genetic variants to protect against autoimmune diseases. The magnitude of the challenge of unveiling the pathogenesis of autoimmune diseases is reinforced by Robino et al. who observed non-classical HLA haplotypes that predispose to type 1 diabetes. However, this enormous challenge begins to be unveiled with the Nizam et al. study presenting the key regulatory link between hsa-miR-320-3p and type 1 diabetes.

The power of genetic variants to drive immunological disorders is also revealed by Adi et al. in a 3-year-old boy with generalized pustular psoriasis and an IL36RN variant, and by Wang et al. in a report of six families with retinopathy and seven pathogenic variants in four different genes.

Similarly, the skewed relationship of autoimmune diseases genotype and phenotype is shown by Paldino et al. in a series of pediatric APECED patients who presented with autoimmune hepatitis.

Noteworthy, different cell subsets such as self-reactive B lymphocytes, effector T lymphocytes, neutrophils, low-density granulocytes, monocytes, and natural killer (NK) cells have also been implicated in the pathogenesis of autoimmune diseases (2, 9). Namely, Vissers et al. revealed lower transitional B-cell and NK-cell counts in patients with aplastic anemia. In parallel, an extrafolicular B cell and peripheral T helper cell expansion, as well as, an altered profile of apoptosis-related proteins were observed by Baxter et al. and Liphaus et al. in patients with juvenile-onset systemic lupus erythematosus. Yet, Parackova et al. describe low-density neutrophils from juvenile idiopathic arthritis patients as primed, degranulated, immature cells with impaired suppressive activities. Lastly, Starosz et al. determined the contribution of Th1, Th17, and Th22 lymphocytes in Graves’ disease.

Another intriguing aspect of autoimmune diseases is that they can occur simultaneously (10). Thus, overt or latent polyautoimmunity is frequently observed in a single patient (10). Like this, Leong et al. reinforce that vitiligo may occur with various other autoimmune diseases such as psoriasis.

Classically, autoantibodies are defined as biomarkers of autoimmune disease diagnosis, monitoring, and prediction (10, 11). The array of available autoantibodies and their relationships with the clinical manifestations has grown lately, as demonstrated by Sapana et al. in an 8-year-old boy with anti-GAD65 antibody-positive autoimmune encephalitis and autoimmune polyendocrine syndrome type II. On the other hand, the role of nonspecific autoantibody-related diseases is debated by the Xu et al. study. Recently, the number of antibody-mediated disorders of the central nervous system (CNS) has gradually risen (2). The relevance of this organ-specific autoimmune disease in childhood is highlighted in the reports of Kang et al. and Li et al.

Especially in childhood, tissue damage in the context of autoimmune diseases can negatively impact the body’s physiological development and functioning (2). This impact is covered by various articles in this Research Topic, particularly by Kurpiewska et al. on the pancreatic β-cell function and by Lupu et al. regarding pulmonary lesions.

However, genetics is not the only component, the epigenetic control of gene expression and environmental triggers also make up the puzzle that leads to autoimmune diseases (12). Taking environmental triggers into account, infections are the first to be considered, but often inconclusively. In this regard, Ha et al. add Mycoplasma pneumoniae infection to the puzzle.

Thus, the challenge of autoimmune diseases in childhood is compounded by the limited arsenal of treatments available for this age group (2, 7). Even with efforts toward precision therapy, response prediction biomarkers, and newly developed biologics such as TNF-α blockers, targeted B-cell therapies, and Janus kinase inhibitors, in addition to classically nonbiologic treatments such as methotrexate and intravenous immunoglobulin, also reported in articles in this Research Topic such as that by Chen et al., the role of glucocorticoid therapy to control autoimmune disease flares persists, as discussed by Cao et al. and Pan et al. in their manuscripts.

Finally, the editors are very grateful to the authors who contributed to this Frontiers in Immunology Research Topic and invite you to read these interesting articles which intended to clarify the understanding of how autoimmune diseases in childhood develop and progress.

## References

[1] Carneiro-Sampaio M Coutinho A. Tolerance and autoimmunity: Lessons at the bedside of primary immunodeficiencies. Adv Immunol. (2007) 95:51–82. doi: 10.1016/S0065-2776(07)95002-6 17869610

[2] Pisetsky DS Schur PH Romain PL. Overview of autoimmunity. (2020), Uptodate. com.

[3] Schmidt RE Grimbacher B Witte T. Autoimmunity and primary immunodeficiency: two sides of the same coin? Nat Rev Rheumatol. (2018) 14:7–18. doi: 10.1038/nrrheum.2017.198 29255211

[4] Santana-Santos D Liphaus BL Beatrice JM Carneiro J DA Carneiro-Sampaio M MS Rangel-Santos A. Low T-cell receptor excision circles (TREC) in children and adolescents with immune thrombocytopenia. Br J Haematol. (2021) 192:e99–e102. doi: 10.1111/bjh.17289 33348432

[5] Liphaus BL Kiss MHB Goldberg AC. HLA-DRB1 alleles in juvenile-onset systemic lupus erythematosus: renal histologic class correlations. Braz J Med Biol Res. (2007) 40:591–7. doi: 10.1590/S0100-879X2007000400019 17401504

[6] Tangye SG Al-Herz W Bousfiha A Cunningham-Rundles C Franco JL Holland SM. Human inborn errors of immunity: 2022 update on the classification from the international union of immunological societies expert committee. J Clin Immunol. (2022) 42:1473–507. doi: 10.1007/s10875-022-01289-3 PMC9244088 35748970

[7] Carneiro-Sampaio M Liphaus BL Jesus AA Silva CAA Oliveira JB Kiss MH. Understanding systemic lupus erythematosus physiopathology in the light of primary immunodeficiencies. J Clin Immunol. (2008) 28:S34–41. doi: 10.1007/s10875-008-9187-2 18404362

[8] Liphaus BL Caramalho I Rangel-Santos A Silva CA Demengeot J Carneiro-Sampaio MMS. LRBA deficiency: a new genetic cause of monogenic lúpus. Ann Rheum Dis. (2020) 79:427–8. doi: 10.1136/annrheumdis-2019-216410 31852671

[9] Liphaus BL Kiss MHB Carrasco S Goldenstein-Schainberg C. Increased fas and bcl-2 expression on peripheral mononuclear cells from patients with active juvenile-onset systemic lupus erythematosus. J Rheumatol. (2007) 34:1580–4. 17516614

[10] Kamioka PE Liphaus BL Beatrice JM Matsumoto LA Reis JMA Lima L. Latent and overt polyautoimmunity in children and adolescents with immune thrombocytopenia. J Ped Hematol Oncol. (2020) 42:e6060–e609. doi: 10.1097/MPH.0000000000001836 32459722

[11] Aikawa NE Jesus AA Liphaus BL Silva CA Carneiro-Sampaio M Viana VT. Organ-specific autoantibodies and autoimmune diseases in juvenile systemic lupus erythematosus and juvenile dermatomyisistis patients. Clin Exp Rheumatol. (2012) 30:126–31. 22261392

[12] Brooks WH Dantec CL Pers JO Youinou P Renaudineau Y. Epigenetics and autoimmunity. J Autoimmun. (2010) 34:j207–9. doi: 10.1016/j.jaut.2009.12.006 20053532

